# Analysis, modeling, control and operation of an interleaved three-port boost converter for DMPPT systems including PV and storage at module level

**DOI:** 10.1016/j.heliyon.2019.e01402

**Published:** 2019-03-21

**Authors:** Ander González, Ramón López-Erauskin, Johan Gyselinck

**Affiliations:** Bio Electro and Mechanical Systems (BEAMS) Department, Université Libre de Bruxelles, 50 Franklin Roosevelt Ave. (CP165/52), B-1050, Brussels, Belgium

**Keywords:** Electrical engineering, Energy engineering

## Abstract

This paper presents the analysis, control and implementation of the interleaved three-port boost converter. The scope of this paper is the interfacing of photovoltaic systems that include storage. A new symmetrical PWM modulation strategy that prevents unwanted switching states without requiring external circuitry is presented. This modulation allows for proper sampling of the measurements, increasing thus their accuracy. Large- and small-signal models of the interleaved and non-interleaved three port boost converters are presented and transfer functions are derived for control design purposes. The different currents in the converter are controlled using control loops that govern the behavior of the converter. These loops are intuitively designed by treating them independently. With the proper loop bandwidth selection, the converter achieves fast response and good reference tracking and is suitable to interface photovoltaic and storage systems with different kinds of loads. The presented models, modulation and control loops are validated through simulation and with experimental results.

## 1 Introduction

In the course of time, more countries are changing their policies to promote renewable energy sources. In 2005, 55 countries had developed policies supporting renewables, while in 2013 the number of countries rose up to 144 [1]. The growing interest in combination of renewable energy sources and energy storage systems demands for more research. Power electronic converters are key to develop efficient solutions that integrate both parts. A way to increase the efficiency of such systems is reducing the power conversion stages in the converter. There are several three-port converter solutions in the literature that aim to interface renewable sources, storage and a load using a single power conversion stage.

Various works present non-isolated topologies [2], [3] that use a single-switch [4], or present different approaches based on classic topologies [5]. In [6] the author uses a mix of buck and boost converter to interface photovoltaic (PV) and battery systems. There is also research on high-voltage gain converters using coupled inductors [7], [8], [9], [10]. Other converters are based on half-bridge topologies [11] and converters for different applications such as automotive can be found [12].

Among the works published using isolated converters the full-bridge topology is very popular [13], [14], [15], [16], [17], [18], [19]. Some authors focus their work on soft-switching [20], [21], [22] while others present control of the converters combining different modulation strategies such as pulse-frequency modulation plus pulse width modulation (PWM) [23], or phase-shift modulation plus PWM [24]. There are other topologies [25], using half-bridges [26] or coupled inductors controlled using phase-shift modulation plus PWM [27].

Some multi-input multi-output converters are described in the literature [28], for wind PV and storage [29], for automotive application including a source, batteries and supercapacitors [30] and boost converter based topologies [31]. Also different methodologies to synthesize three-port converters have been published [32], [33], [34], [35], [36], [37]. In [38] the control of the converters presented in [32] is discussed. Different extensive reviews on multi-input converters can be found in the literature, for renewable applications [39], hybrid vehicle applications [40] and distributed generation units [41]. A review on three-port converters for the integration of renewable energy and energy storage system can be found in [42]. Also a review of high-voltage gain DC/DC converters for photovoltaic applications can be found in [43].

Many of the papers present control operation that depends on the working mode of the converter, i.e. on the port(s) that is/are supplying and receiving power. In this paper a control that is valid regardless the working mode of the converter is presented along with other work done on the topology presented in [44] that is derived from [32], [33]. As stated in [32], [33], this converter topology allows for the reduction of magnetic components when compared to the use of separated boost converters.

**Figure 1:**
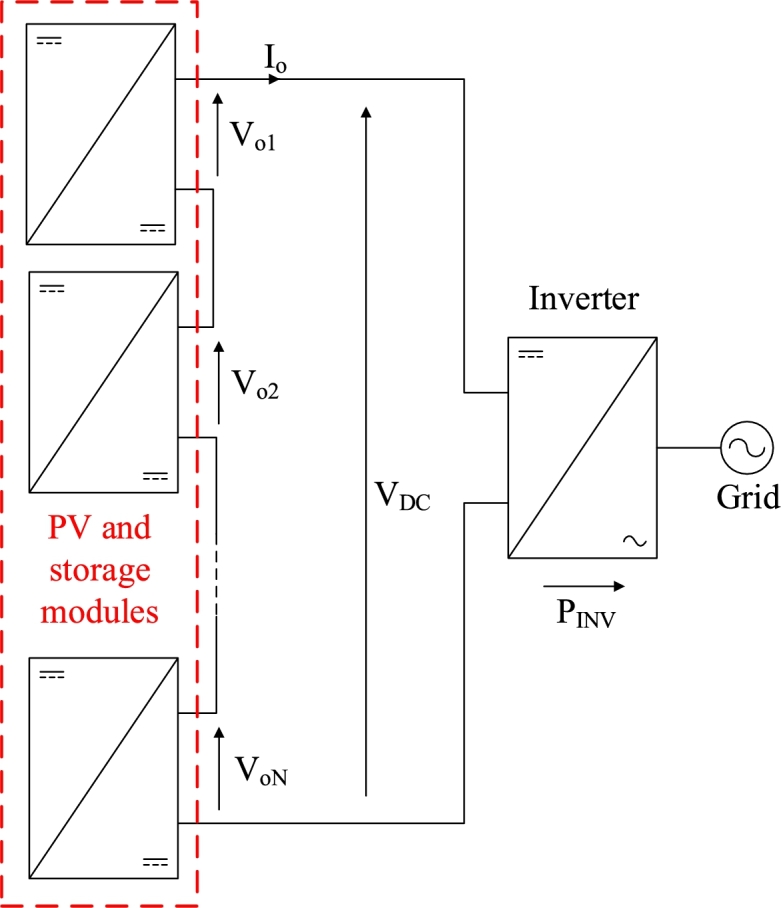
Target distributed MPPT application topology, including *N* number of modules and connected to the grid by means of an inverter.

The final purpose of the system is to build a distributed maximum power point tracking (MPPT) system including storage at module level as shown in Figure 1 and presented in [45], [46]. These systems include several modules as the one presented here in series to form a high-voltage string of so called *optimizers* that are connected to a DC-bus. Such systems aim to increase the production of PV energy by performing the MPPT at PV panel level. This overcomes power loss arising from different factors, such as dust deposition [47], partial shading, different orientations, manufacturing tolerances and uneven aging of the panels [48]. Since these converters are connected in series the output voltage ripple of each module adds to the others. For this reason a very low voltage ripple is pursued. The boost-TPC [32], [33] was designed for standalone operation and may reach high voltage and current ripples when high power is demanded [44]. Interleaving of converters has proven to be useful in improving conversion efficiency, reducing current and voltage ripples and shrinking capacitor and inductor sizes [49].

There are several considerations attached to the target application. First, as it is a grid-tied system, a minimum DC-bus voltage is required for the inverter to work. For this reason, voltage boosting topologies are only considered. In order to extend battery lifetime, converter topologies that are able to completely restrict the current flow through the battery when this is not used are desired. A useful and desired feature, is that the DC/DC converter in the module is able to keep the PV voltage in its output when the converter is off. This allows for turning off the modules and controlling the whole system using solely the inverter, as in classical PV installations. Besides, when modules are turned on or off, the difference in one module's output voltage has to be compensated by the rest of the modules in the string. This means that when a module is turned off, the output voltage of the module will usually drop. If the voltage drops to the PV voltage instead of zero, the effect on the rest of the modules in the same string will be lower. As each of the PV panels is tied to a module, the price of the converter in the modules needs to be low. For this reason, topologies including a large number of components, coupled inductors or high-frequency transformers are discarded. Many of the topologies presented in literature require complex control strategies, some of which need to be changed depending on the power-flow in the converter. Here a simple control structure is pursued. All of the cited topologies fail in meeting one or more of the mentioned desired features. For this reason the topology and control described in this paper are proposed for the storage including distributed MPPT system presented.

In this paper, interleaving improved the battery current ripple, which leads to increased battery life making the system useful for renewable grid-tied systems including energy storage. Moreover, this also decreased the output voltage ripple, an important concern in series connected distributed MPPT systems. Besides, an improved modulation strategy that avoids unwanted states and that is suitable for both non-interleaved and interleaved three-port boost converters is introduced in this paper. In order to simulate and control the presented system, averaged and small-signal models valid for both non-interleaved and interleaved three-port boost converters are presented. These models describe the system in any of its working modes without the need of swapping transfer functions as in [32], [33].

## 2 Analysis

The interleaved three-port boost converter is able to transfer power from PV port to battery and output ports, and from battery port to output port. As it works in the way classical boost converters do, the same voltage and current requirements apply, $V_{o}>V_{b}>V_{\mathrm{PV}}$ and $I_{\mathrm{in}}>I_{o}$ where $I_{\mathrm{in}}=\left(1-d_{3}\right)I_{\mathrm{PV}}+d_{3}I_{b}$ is the equivalent current derived from PV and battery input currents. If the battery is not used, the restriction $V_{o}>V_{b}$ can be overridden.

**Figure 2:**
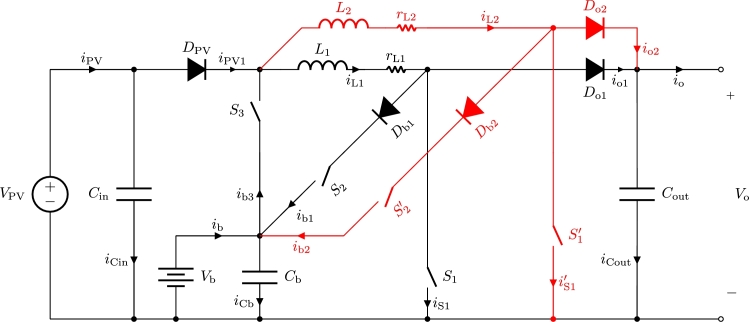
Interleaved three-port boost converter topology with interleaving branch in red. Converter includes unidirectional PV and output ports and a bidirectional battery port.

One of the salient features of the interleaved three-port boost converter is its simplicity. In essence, this topology works like a classic boost converter which has the ability to swap its inputs and outputs. As described in Figure 2 the input of the converter is $V_{\mathrm{PV}}$ as long as $S_{3}$ is open, whilst $V_{b}$ supplies the input port when $S_{3}$ is closed. The input power supply is thus controlled using the switch $S_{3}$ that will provoke $D_{\mathrm{PV}}$ to be forward- or reverse-biased. In a similar manner the output power flow is controlled using switches $S_{2}$ and $S_{2}^{\prime }$. When $S_{2}$ ($S_{2}^{\prime }$) is on, the output diode $D_{\mathrm{o1}}$ ($D_{\mathrm{o2}}$) is reverse-biased and the battery port acts as output of the boost converter. When $S_{2}$ ($S_{2}^{\prime }$) is off, the output diode $D_{\mathrm{o1}}$ ($D_{\mathrm{o2}}$) is forward-biased and the power flows to the output port charging $C_{\mathrm{out}}$. $D_{\mathrm{b1}}$ and $D_{\mathrm{b2}}$ diodes are included in order to avoid undesired current-flow through the body-diodes of switches $S_{2}$ and $S_{2}^{\prime }$ respectively.

The total transferred power is proportional to $i_{\mathrm{L1}}$ and $i_{\mathrm{L2}}$ inductor currents. Charge and discharge of inductors $L_{1}$ and $L_{2}$ are controlled using switches $S_{1}$ and $S_{1}^{\prime }$ respectively. Therefore duty cycles $d_{1}$ and $d_{1}^{\prime }$ that are applied to switches $S_{1}$ and $S_{1}^{\prime }$ respectively, control the total power transfer at each moment.

In this topology the simultaneous ON-state in switches $S_{1}$ and $S_{2}$ (or $S_{1}^{\prime }$ and $S_{2}^{\prime }$) is to be avoided. Although it is not destructive for the converter, when $S_{1}$ and $S_{2}$ ($S_{1}^{\prime }$ and $S_{2}^{\prime }$) are on at the same time, the dominant behavior will be the one pursued by closing $S_{1}$ ($S_{1}^{\prime }$), letting the inductor charge while not supplying any current to charge the battery as closing of $S_{2}$ ($S_{2}^{\prime }$) would intend. If this occurs, the models derived in the next section will not describe accurately the converter behavior. This problem was addressed in [33] using external circuitry for the boost three-port converter. The novel modulations presented here avoid the requirement for external circuitry by means of proper timing.

**Figure 3:**
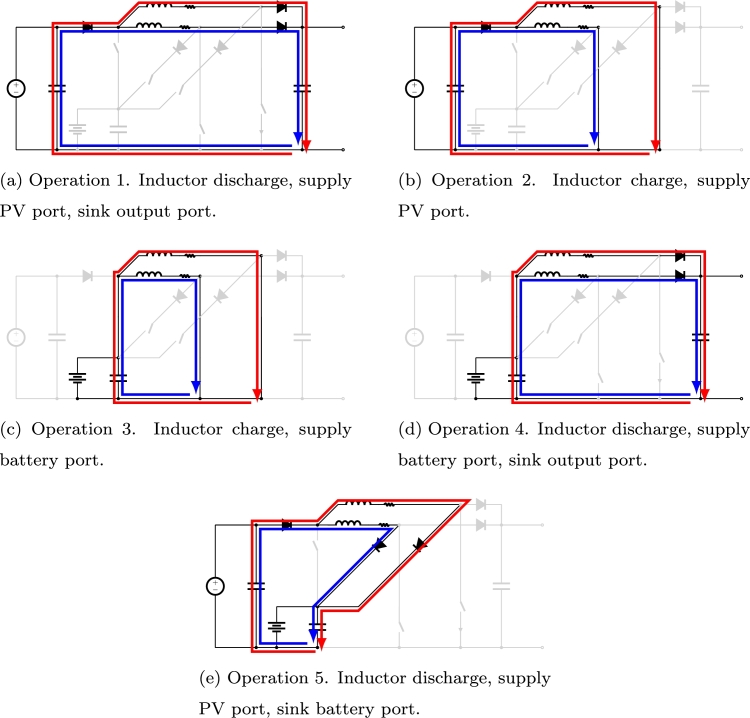
Main operation modes of the interleaved three-port boost converter. In blue the current of the first branch of the converter, in red the current of the second branch of the converter.

In Figure 3 current paths of both branches are represented for different operations in the converter. Both currents, red and blue, do not necessarily occur at the same time. Whether or not these two currents appear simultaneously, will be the result of the switching functions at each moment. In Figure 3a the inductors are discharged while PV port supplies power to the output. In Figure 3b and 3c the inductors are charged using PV and battery power respectively. In Figure 3d the inductors are discharged while battery port supplies power to the output. Finally, in Figure 3e the inductors are discharged while PV port supplies power to the battery.

Thanks to the described operation modes, the interleaved three-port boost converter is able to transfer energy from and to the different ports using a single power conversion path, reducing thus conversion-stages when compared to other topologies.

## 3 Model

In [32], [33] different transfer functions to control the output voltage are presented. The control needs to swap from one transfer function to another depending on which port supplies or sinks the power transferred. Here the switched, averaged and small-signal models are presented that are valid for any of the operation modes presented in section 2. The small-signal model is a useful tool derived from the averaged model that linearizes the behavior of the converter around an operating point. This model lets the control designer to deduce the required transfer functions to control any voltage or current of the converter.

All models presented in this section are useful to describe the behavior of the boost-TPC converter presented in [32], [33]. In order to do so, the terms corresponding to interleaving branch (red branch in Figure 2) have to be ignored.

### 3.1 Switched model

A generally valid model based on the current flow can be developed looking at the voltages across inductors $L_{1}$ and $L_{2}$:

(1) $$L_{1}\frac{di_{\text{L}1}}{dt}=-r_{\mathrm{L1}}i_{\mathrm{L1}}+{\bar{u}}_{3}V_{\text{PV}}+u_{3}V_{\text{b}}-{\bar{u}}_{1}{\bar{u}}_{2}V_{\text{o}}-{\bar{u}}_{1}u_{2}V_{\text{b}}$$

(2) $$L_{2}\frac{di_{\text{L2}}}{dt}=-r_{\mathrm{L2}}i_{\mathrm{L2}}+{\bar{u}}_{3}V_{\text{PV}}+u_{3}V_{\text{bat}}-{\bar{u}}_{1}^{\prime }{\bar{u}}_{2}^{\prime }V_{\text{o}}-{\bar{u}}_{1}^{\prime }u_{2}^{\prime }V_{\text{bat}}$$

where $i_{\mathrm{L1}}$ and $i_{\mathrm{L2}}$ are the current in inductors $L_{1}$ and $L_{2}$ respectively, $u_{1}$ to $u_{3}$ the switching functions of the corresponding switches (instantaneous value equal to either 1 or 0, when the switch is closed or open respectively), and ${\bar{u}}_{1}$ to ${\bar{u}}_{3}$ the complementary of the corresponding switching functions.

The currents $i_{\mathrm{L1}}$ and $i_{\mathrm{L2}}$ can be directed to the different ports selecting the corresponding switches as follows:

(3) $$i_{\text{PV1}}={\bar{u}}_{3}\left(i_{\text{L1}}+i_{\mathrm{L2}}\right)$$

(4) $$i_{\text{b}}-i_{\mathrm{Cb}}=\left(u_{3}-u_{2}\right)i_{\text{L1}}+\left(u_{3}-u_{2}^{\prime }\right)i_{\text{L2}}$$

(5) $$i_{\mathrm{Cout}}={\bar{u}}_{1}{\bar{u}}_{2}i_{\text{L1}}+{\bar{u}}_{1}^{\prime }{\bar{u}}_{2}^{\prime }i_{\text{L2}}-i_{o}$$

This set of four equations describes the behavior of the converter in any of its working modes. The three capacitor voltages are obtained through the integration of the current of the corresponding port as follows:

(6) $$v_{\mathrm{PV}}=V_{\mathrm{PV0}}+\frac{1}{C_{\mathrm{in}}}\int i_{\mathrm{Cin}}dt$$

(7) $$v_{b}=V_{\mathrm{b0}}+\frac{1}{C_{b}}\int i_{\mathrm{Cb}}dt$$

(8) $$v_{o}=V_{\mathrm{o0}}+\frac{1}{C_{\mathrm{out}}}\int i_{\mathrm{Cout}}dt$$

where $V_{\mathrm{PV0}}$, $V_{\mathrm{b0}}$ and $V_{\mathrm{o0}}$ are the initial voltages in the PV, battery and output ports respectively.

### 3.2 Averaged model

Using the detailed model of the interleaved three-port boost converter [44] and applying an averaging time-window width equal to the switching periods of the switches the following averaged model is obtained:

(9) $$L_{1}\frac{dI_{L1}}{dt}=-r_{\mathrm{L1}}I_{\mathrm{L1}}+\left(1-d_{3}\right)V_{\mathrm{PV}}+d_{3}V_{b}-\left(1-d_{1}-d_{2}\right)V_{o}-d_{2}V_{b}$$

where the current $I_{\mathrm{L1}}$ is the average value of the current through the inductor $L_{1}$ and $d_{1}$ to $d_{3}$ the duty cycles of the corresponding switches. In the same way the equation for inductor $L_{2}$ is obtained:

(10) $$L_{2}\frac{dI_{\mathrm{L2}}}{dt}=-r_{\mathrm{L2}}I_{\mathrm{L2}}+\left(1-d_{3}\right)V_{\mathrm{PV}}+d_{3}V_{b}-\left(1-d_{1}^{\prime }-d_{2}^{\prime }\right)V_{o}-d_{2}^{\prime }V_{b}$$

where the current $I_{\mathrm{L2}}$ is the average value of the current through the inductor $L_{2}$ and $d_{1}^{\prime }$ and $d_{2}^{\prime }$ the duty cycles of the corresponding switches. The currents $I_{\mathrm{L1}}$ and $I_{\mathrm{L2}}$ can be diverted to the different ports selecting the corresponding duty cycles:

(11) $$I_{\mathrm{PV1}}=\left(1-d_{3}\right)\left(I_{\mathrm{L1}}+I_{\mathrm{L2}}\right)$$

(12) $$I_{b}-I_{\mathrm{Cb}}=\left(d_{3}-d_{2}\right)I_{\mathrm{L1}}+\left(d_{3}-d_{2}^{\prime }\right)I_{\mathrm{L2}}$$

(13) $$I_{\mathrm{Cout}}=\left(1-d_{1}-d_{2}\right)I_{\mathrm{L1}}+\left(1-d_{1}^{\prime }-d_{2}^{\prime }\right)I_{\mathrm{L2}}-I_{o}$$

This set of five equations describes the behavior of the converter in any of its working modes. Capacitor voltages are obtained through the integration of the current of the corresponding port.

### 3.3 Small-signal model

Assuming that all the port voltages are constant and disturbance free, the following small-signal model can be derived perturbing and linearizing the inductor and port currents, output voltage and all duty cycles. Perturbed variables are replaced by $x=\overset{ˆ}{x}+X$ where *x* is the variable to be perturbed, $\overset{ˆ}{x}$ is the perturbation applied to the variable and *X* is the DC value of the variable at the linearization point.

(14) $$\begin{array}{c} L_{1}\frac{d{\overset{ˆ}{i}}_{\mathrm{L1}}}{dt}=-r_{\mathrm{L1}}{\overset{ˆ}{i}}_{\mathrm{L1}}-\left(1-D_{1}-D_{2}\right){\overset{ˆ}{v}}_{o}+V_{o}{\overset{ˆ}{d}}_{1}\\ +\left(V_{o}-V_{b}\right){\overset{ˆ}{d}}_{2}+\left(V_{b}-V_{\mathrm{PV}}\right){\overset{ˆ}{d}}_{3} \end{array}$$

(15) $$\begin{array}{c} L_{2}\frac{d{\overset{ˆ}{i}}_{\mathrm{L2}}}{dt}=-r_{\mathrm{L2}}{\overset{ˆ}{i}}_{\mathrm{L2}}-\left(1-D_{1}^{\prime }-D_{2}^{\prime }\right){\overset{ˆ}{v}}_{o}+V_{o}{\overset{ˆ}{d}}_{1}^{\prime }\\ +\left(V_{o}-V_{b}\right){\overset{ˆ}{d}}_{2}^{\prime }+\left(V_{b}-V_{\mathrm{PV}}\right){\overset{ˆ}{d}}_{3} \end{array}$$

(16) $$\begin{array}{c} C_{\mathrm{out}}\frac{d{\overset{ˆ}{v}}_{o}}{dt}=\left(1-D_{1}-D_{2}\right){\overset{ˆ}{i}}_{\mathrm{L1}}+\left(1-D_{1}^{\prime }-D_{2}^{\prime }\right){\overset{ˆ}{i}}_{\mathrm{L2}}\\ -I_{\mathrm{L1}}{\overset{ˆ}{d}}_{1}-I_{\mathrm{L2}}{\overset{ˆ}{d}}_{1}^{\prime }-I_{\mathrm{L1}}{\overset{ˆ}{d}}_{2}-I_{\mathrm{L2}}{\overset{ˆ}{d}}_{2}^{\prime } \end{array}$$

(17) $${\overset{ˆ}{i}}_{\mathrm{PV1}}=\left(1-D_{3}\right)\left({\overset{ˆ}{i}}_{\mathrm{L1}}+{\overset{ˆ}{i}}_{\mathrm{L2}}\right)-\left(I_{\mathrm{L1}}+I_{\mathrm{L2}}\right){\overset{ˆ}{d}}_{3}$$

(18) $${\overset{ˆ}{i}}_{b}=\left(D_{3}-D_{2}\right){\overset{ˆ}{i}}_{\mathrm{L1}}+\left(D_{3}-D_{2}^{\prime }\right){\overset{ˆ}{i}}_{\mathrm{L2}}-I_{\mathrm{L1}}{\overset{ˆ}{d}}_{2}-I_{\mathrm{L2}}{\overset{ˆ}{d}}_{2}^{\prime }+\left(I_{\mathrm{L1}}+I_{\mathrm{L2}}\right){\overset{ˆ}{d}}_{3}$$

(19) $${\overset{ˆ}{i}}_{\mathrm{o1}}=\left(1-D_{1}-D_{2}\right){\overset{ˆ}{i}}_{\mathrm{L1}}-I_{\mathrm{L1}}{\overset{ˆ}{d}}_{1}-I_{\mathrm{L1}}{\overset{ˆ}{d}}_{2}$$

(20) $${\overset{ˆ}{i}}_{\mathrm{o2}}=\left(1-D_{1}^{\prime }-D_{2}^{\prime }\right){\overset{ˆ}{i}}_{\mathrm{L2}}-I_{\mathrm{L2}}{\overset{ˆ}{d}}_{1}^{\prime }-I_{\mathrm{L2}}{\overset{ˆ}{d}}_{2}^{\prime }$$

If $D_{1}=D_{1}^{\prime }$, ${\overset{ˆ}{d}}_{1}={\overset{ˆ}{d}}_{1}^{\prime }$, $D_{2}=D_{2}^{\prime }$, ${\overset{ˆ}{d}}_{2}={\overset{ˆ}{d}}_{2}^{\prime }$, $L_{1}=L_{2}=L$ and $r_{\mathrm{L1}}=r_{\mathrm{L2}}=r_{L}$ is assumed, it results that $I_{\mathrm{L1}}=I_{\mathrm{L2}}$, ${\overset{ˆ}{i}}_{\mathrm{L1}}={\overset{ˆ}{i}}_{\mathrm{L2}}={\overset{ˆ}{i}}_{L}$ and ${\overset{ˆ}{i}}_{\mathrm{o1}}={\overset{ˆ}{i}}_{\mathrm{o2}}={\overset{ˆ}{i}}_{\mathrm{out}}$ holds true. Using these assumptions greatly simplifies the small-signal model and among others, the following interesting transfer functions are found:

(21) $$G_{\mathrm{L1}}=\frac{{\overset{ˆ}{i}}_{L}}{{\overset{ˆ}{d}}_{1}}=\frac{sC_{\mathrm{out}}V_{o}+2\left(1-D_{1}-D_{2}\right)I_{L}}{s^{2}LC_{\mathrm{out}}+sC_{\mathrm{out}}r_{L}+2{\left(1-D_{1}-D_{2}\right)}^{2}}$$

(22) $$G_{\mathrm{L2}}=\frac{{\overset{ˆ}{i}}_{L}}{{\overset{ˆ}{d}}_{2}}=\frac{sC_{\mathrm{out}}\left(V_{o}-V_{b}\right)+2\left(1-D_{1}-D_{2}\right)I_{L}}{s^{2}LC_{\mathrm{out}}+sC_{\mathrm{out}}r_{L}+2{\left(1-D_{1}-D_{2}\right)}^{2}}$$

(23) $$G_{\mathrm{L3}}=\frac{{\overset{ˆ}{i}}_{L}}{{\overset{ˆ}{d}}_{3}}=\frac{sC_{\mathrm{out}}\left(V_{b}-V_{\mathrm{PV}}\right)}{s^{2}LC_{\mathrm{out}}+sC_{\mathrm{out}}r_{L}+2{\left(1-D_{1}-D_{2}\right)}^{2}}$$

(24) $$G_{\mathrm{PV1}}=\frac{{\overset{ˆ}{i}}_{\mathrm{PV1}}}{{\overset{ˆ}{i}}_{L}}=2\left(1-D_{3}\right)$$

(25) $$G_{\mathrm{b2}}=\frac{{\overset{ˆ}{i}}_{b}}{{\overset{ˆ}{d}}_{2}}=-2I_{L}$$

(26) $$G_{\mathrm{b3}}=\frac{{\overset{ˆ}{i}}_{b}}{{\overset{ˆ}{d}}_{3}}=2I_{L}$$

**Figure 4:**
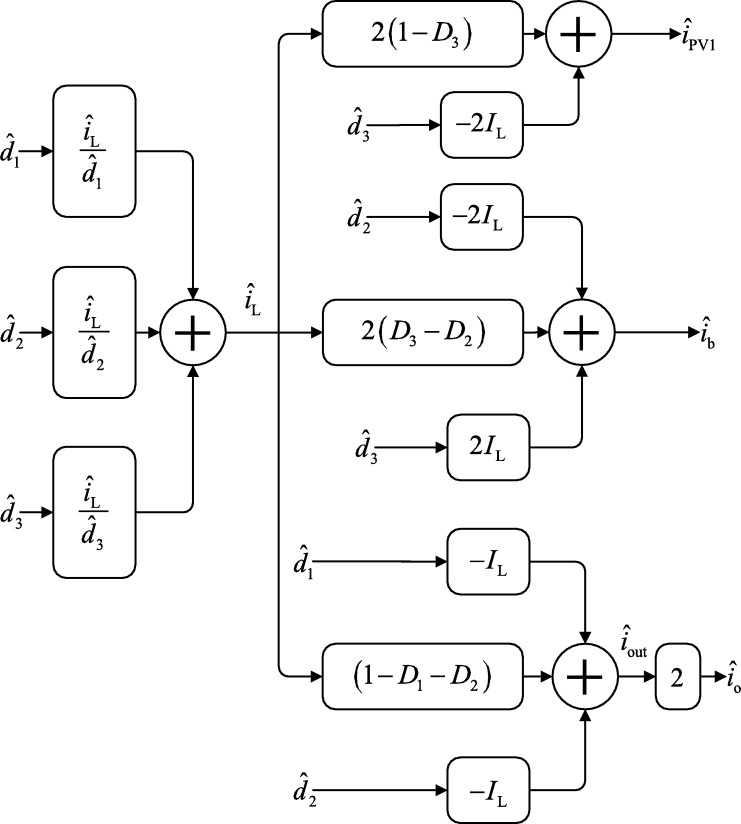
Diagram block representation of the converter using transfer functions derived from presented small-signal model.

In Figure 4 the derived small-signal model is represented using diagram blocks.

## 4 Methods

Here the proposed modulation strategies and control loops to operate the converter are presented. These are used in order to achieve proper converter operation, controlling the currents and voltages in the converter.

### 4.1 Proposed modulation

In [33] a solution to avoid undesired simultaneous ON-states of the switches is presented. This consists in a number of comparators and logic gates and works for the non-interleaved three-port boost converter. In [44] a modulation scheme that overcomes the simultaneous switching issue without external circuitry is presented. That modulation scheme relays in trailing- and leading-edge carriers and is suitable for interleaved and non-interleaved converters. Here, that modulation scheme is explained and a new modulation scheme that uses symmetrical carriers is presented and compared to the trailing- and leading-edge modulation. The use of symmetrical carriers is more suitable for digital implementation of the system, allowing for proper scheduling of the analog-to-digital converter (ADC) sampling. This new modulation scheme does not require any external circuit as the trailing- and leading-edge modulation, improving the scheduling of the ADC sampling while reducing the number of required carriers.

#### 4.1.1 Trailing- and leading-edge modulation

This converter modulation was presented in [44] and uses five different synchronized PWM modules. $S_{1}$ and $S_{1}^{\prime }$ driving modules consist in an up-counting sawtooth carrier (trailing-edge modulation) with frequency $f_{\mathrm{sw}}$ that is compared to their corresponding reference waveform, that are $D_{1}$ and $D_{1}^{\prime }$ respectively. $S_{2}$ and $S_{2}^{\prime }$ driving modules consist in a down-counting sawtooth carrier (leading-edge modulation) with frequency $f_{\mathrm{sw}}$ that is compared to their corresponding reference waveform, that are $D_{2}$ and $D_{2}^{\prime }$ respectively. $S_{3}$ driving module consists in an up-counting sawtooth carrier with frequency $2f_{\mathrm{sw}}$ that is compared to $D_{3}$ reference waveform.

PWM modules driving $S_{1}^{\prime }$ and $S_{2}^{\prime }$ switches include a phase-shift of 180° so as to reduce the voltage and current ripples in the different ports of the converter.

All duty cycles $D_{1}$, $D_{1}^{\prime }$, $D_{2}$, $D_{2}^{\prime }$ and $D_{3}$ are continuous quantities that range from 0 to 1, both included. The comparison of the duty cycle $D_{x}$, to its corresponding carrier $c_{x}$, results in the gate pulses $u_{x}$ that are applied to the gate of the corresponding MOSFET $S_{x}$.

$D_{2}$ and $D_{2}^{\prime }$ are nonzero when the battery is charged. On the other hand, when the battery is discharged, $D_{3}$ will be nonzero. Therefore there will not be a case where $D_{2}$ and $D_{2}^{\prime }$ are greater than zero at the same time as $D_{3}$ is greater than zero. Thanks to that, it is not required to check if the duty cycles coming from the control are appropriate in this case. However, it is necessary to check if the duty cycles $D_{1}$ and $D_{2}$ are appropriate for each other, this applies to $D_{1}^{\prime }$ and $D_{2}^{\prime }$ as well. Thanks to the up- and down-counting carriers, avoiding simultaneous ON-states is addressed by checking the sum of their corresponding duty cycles. If the sum $D_{1}+D_{2}<1$ the converter is working as expected, while $D_{1}+D_{2}>1$ means that an overlap of $S_{1}$ and $S_{2}$ ON-states is occurring.

**Figure 5:**
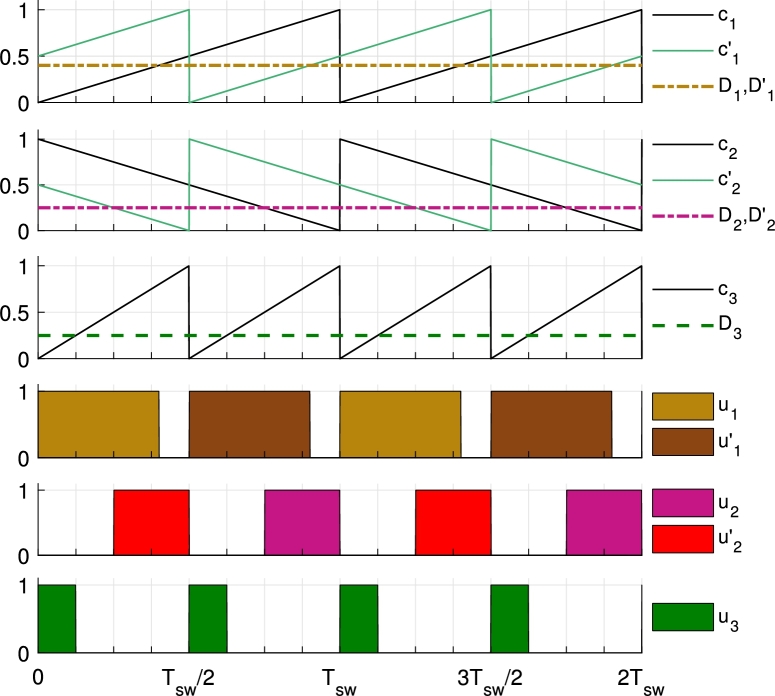
Up- and down-counting modulation (trailing- and leading-edge modulation) to avoid simultaneous ON-states of *S*_1_ and *S*_2_ (and $S_{1}^{\prime }$ and $S_{2}^{\prime }$). Proposed in [44].

An example of the switching signals of this modulation is shown in Figure 5.

#### 4.1.2 Symmetrical modulation

This new modulation reduces the number of carriers by 2 when compared to the trailing- and leading-edge modulation presented in [44]. It requires the use of a carrier with phase 0° and frequency $f_{\mathrm{sw}}$ ($c_{1}$), a carrier with phase 180° and frequency $f_{\mathrm{sw}}$ ($c_{2}$) and a carrier with phase 0° and frequency $2f_{\mathrm{sw}}$ ($c_{3}$). In order to get similar results to the ones obtained using the trailing- and leading-edge modulation, $D_{1}$ and $D_{2}^{\prime }$ are compared to $c_{1}$, $D_{1}^{\prime }$ and $D_{2}$ are compared to $c_{2}$ and $D_{3}$ is compared to $c_{3}$. Thanks to the rearrangement of the duty cycle and carrier comparisons and the phase-shift introduced in the carrier $c_{2}$, avoiding simultaneous ON-states in $S_{1}$ and $S_{2}$ (and $S_{1}^{\prime }$ and $S_{2}^{\prime }$) can be addressed by checking the sum $D_{1}+D_{2}$ ($D_{1}^{\prime }+D_{2}^{\prime }$). If the sum does not exceed the value 1, there is no overlapping between $S_{1}$ and $S_{2}$ ($S_{1}^{\prime }$ and $S_{2}^{\prime }$) ON-states.

**Figure 6:**
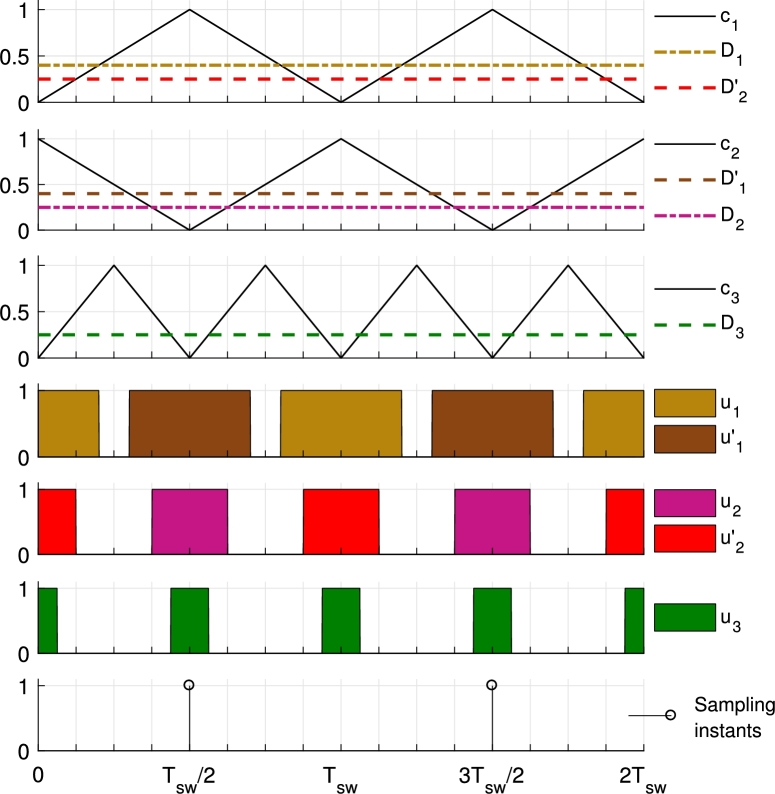
Proposed symmetrical modulation to avoid simultaneous ON-states of *S*_1_ and *S*_2_ (and $S_{1}^{\prime }$ and $S_{2}^{\prime }$) with suggested ADC sampling instants.

Main advantages of this modulation respect to trailing- and leading-edge modulation are (i) the reduced number of carrier signals required, (ii) the ability to sample all the signals at instants without any switching event happening and (iii) the ability to measure the average value of the inductor current. Advantage (i) reduces the number of gates required in FPGA implementation of the modulator whilst the last two enumerated advantages are very useful in any case of digital implementation of the control and are achieved sampling when $c_{1}=1$ as shown in Figure 6 or alternatively, when $c_{1}=0$.

**Figure 7:**
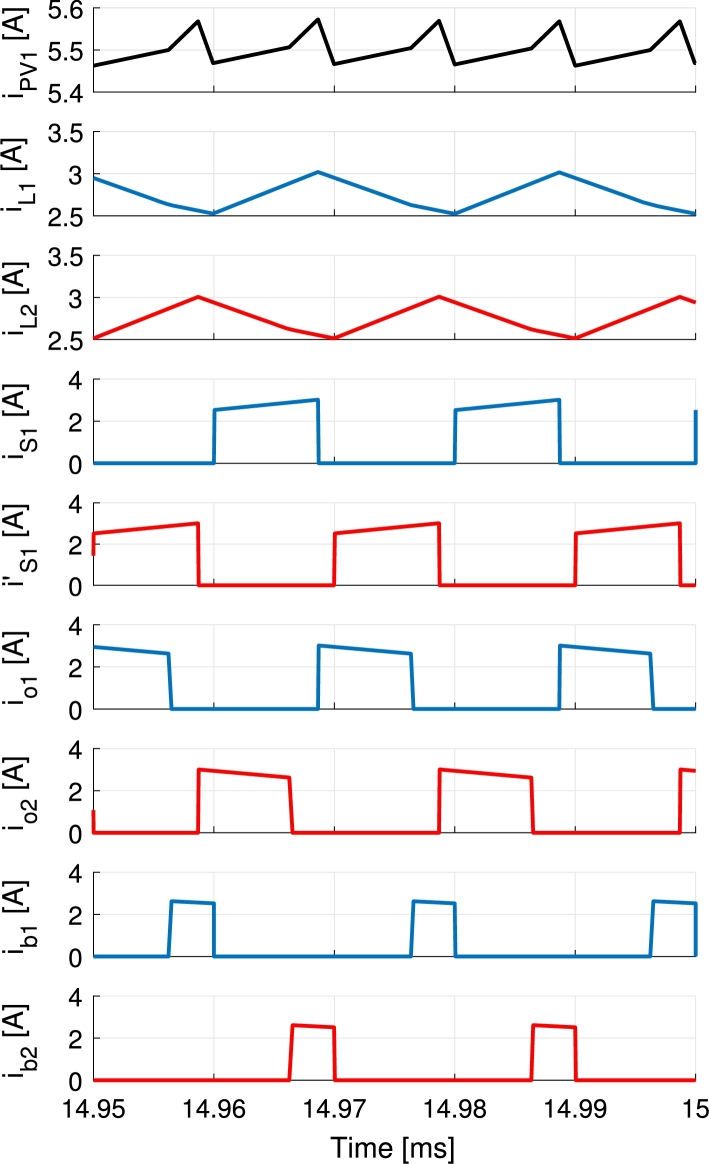
Simulation of the trailing- and leading-edge modulation during battery charge. The currents of the first branch are represented in blue, second branch in red and the waveforms concerning both branches in black.

**Figure 8:**
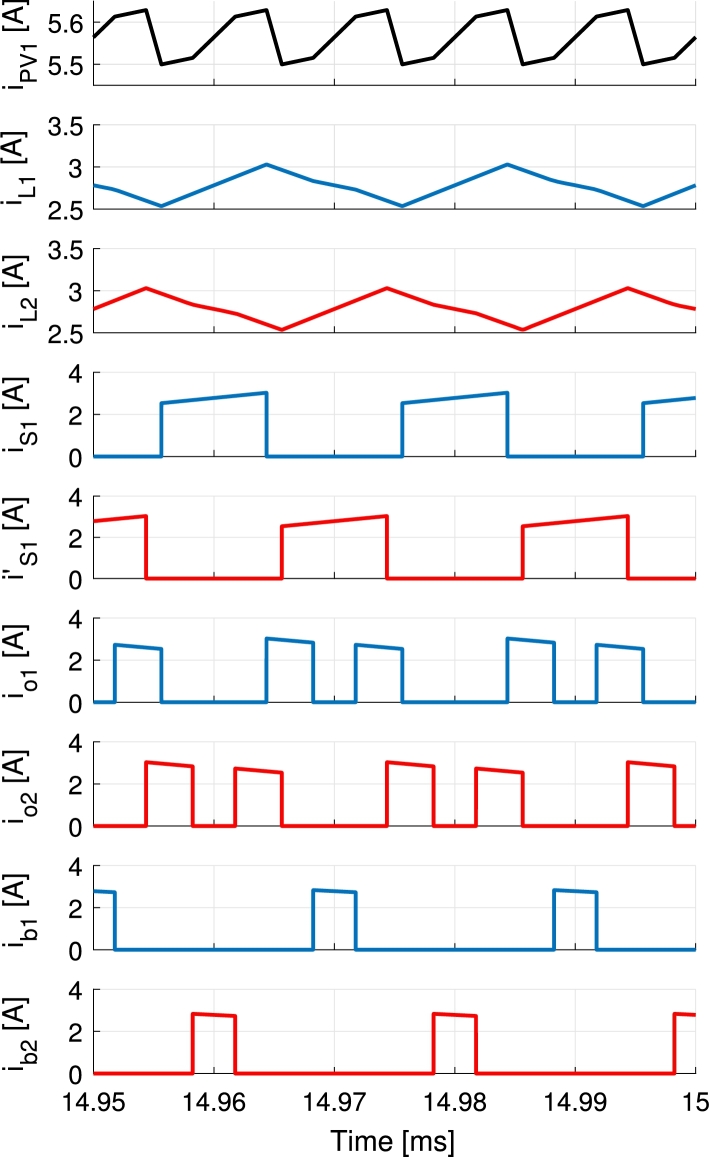
Simulation of the symmetrical modulation during battery charge. The currents of the first branch are represented in blue, second branch in red and the waveforms concerning both branches in black.

A comparison between trailing- and leading-edge modulation and the symmetrical modulation is shown for $I_{\mathrm{PV}}=5.5$ A and $I_{b}=-1$ A in Figures 7 and 8 respectively. These are simulation results for $V_{\mathrm{PV}}=32$ V, $V_{b}=48$ V and $V_{o}=60$ V during steady-state. Experimental result for the symmetrical case is presented later in the text in Figure 18.

### 4.2 Proposed control

Using the small-signal model, the required transfer functions are obtained to design appropriate current and voltage controllers. Cascaded control loops offer over-current protection during start-up and load changes as well as improved response to disturbances [50]. If a nonlinear control that works in extended range is required, different approaches such as feedback linearization have to be used. An example of feedback linearization applied to this topology can be found in [51].

**Figure 9:**
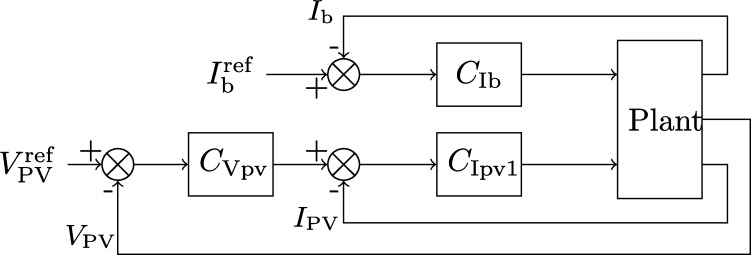
Proposed control scheme with cascaded PV voltage (*C*_Vpv_) and current (*C*_Ipv1_) loops and independent battery current control (*C*_Ib_).

The proposed control, shown in Figure 9, treats separately the PV and battery loops. When PV current reference is different from zero, $D_{1}$ (and $D_{1}^{\prime }$) are controlled by the PV current loop and $D_{2}$ (and $D_{2}^{\prime }$) and $D_{3}$ are controlled by the battery current loop. When PV current reference is equal to zero, battery cannot be charged ($D_{2}=D_{2}^{\prime }=0$) and $D_{1}$ (and $D_{1}^{\prime }$) are controlled by the battery (discharge) loop while $S_{3}$ is kept on all times ($D_{3}=1$). Since the PWM gain corresponding to the switch $S_{3}$ is the double of the other PWM module gains in the latter case the duty cycle is multiplied times 2 in order to keep the same dynamics.

The bandwidth of the controllers is designed to have a sluggish response in all the cases, but the PV current. This design is adopted in order to avoid rapid output power changes. Since the modules in the distributed MPPT system are series connected, rapid output power changes in a module may cause instabilities in the whole string of converters.

#### 4.2.1 PV current control loop

PV current control loop is in charge of controlling the duty cycle of the switch $S_{1}$ (and $S_{1}^{\prime }$) when PV current reference is greater than 0. This means that is responsible of controlling the whole power transmission in the converter and not only the PV current. When a change in battery current is required, the duty cycle of $S_{1}$ (and $S_{1}^{\prime }$) needs to adapt in order to supply the new battery current while keeping the PV current unaltered. For this reason, this loop is designed to be the fastest loop in the system and react rapidly to changes in the other duty cycles.

An average current mode control is chosen. Average current mode control exhibits a higher loop gain at low frequencies and improved noise immunity when compared to current peak mode control [50].

Controller consists in a high-frequency pole to introduce a roll-off of −20 dB/dec that acts as a filter and a PI type controller (type II). The high-frequency pole is placed at a frequency 5 times smaller than the switching frequency, ensuring good filtering of the switching ripple in the current. The PI controller gains are calculated to achieve a crossover frequency higher than 2 kHz and a phase margin of 60° around the operating point described in Table 1 using the component values in Table 2. Using a PWM module gain of $G_{\mathrm{PWM}}=1/1800$ and a sensor gain of $G_{\mathrm{sensor}}=149$, we obtain the uncompensated open-loop gain $T_{u}=G_{\mathrm{L1}}G_{\mathrm{PV1}}G_{\mathrm{PWM}}G_{\mathrm{sensor}}$.

**Table 1:** Selected operating point for PV current-loop control design.

PV current loop	Value
PV power, *P*_PV_	350 W
PV voltage, *V*_PV_	32 V
PV current, *I*_PV_	*I*_PV_ = *P*_PV_/*V*_PV_ = 10.93 A
Output voltage, *V*_o_	60 V
Steady-state *D*_2_ and *D*_3_	0
Steady-state *D*_1_	$D_{1}=1-\frac{\left(1-D_{3}\right)V_{\mathrm{PV}}+D_{3}V_{b}}{\left(1-D_{2}\right)V_{o}+D_{2}V_{b}}=0.467$
Inductor current	$I_{L}=\frac{I_{\mathrm{PV}}}{2\left(1-D_{3}\right)}=5.47$ A

**Table 2:** Selected power converter component values.

Component	Symbol	Value
Inductor	*L*_1_, *L*_2_	560 μH
Input capacitor	*C*_in_	100 μF
Battery port capacitor	*C*_b_	100 μF
Output capacitor	*C*_o_	1000 μF

After placing the compensator zero properly the desired crossover frequency and gain margin are achieved using the following compensator:

(27) $$C_{\mathrm{Ipv}}=\frac{2\pi 10^{4}}{s+2\pi 10^{4}}0.7727\left(1+\frac{2\pi 718}{s}\right)$$

**Figure 10:**
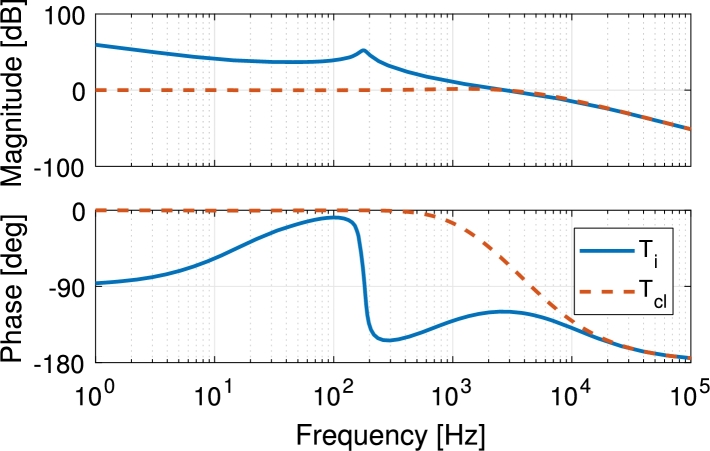
Calculated frequency response of the open- (solid blue) and closed-loop (dashed orange) compensated PV current-loop.

The compensated loop-gain $T_{i}$ achieved a crossover frequency of $f_{c-\mathrm{PV}}=2.72$ kHz and a phase margin of 60.2° using this compensator. The calculated frequency response of the compensated loop and the calculated closed-loop response $T_{\mathrm{cl}}=T_{i}/(1+T_{i})$ are shown in Figure 10.

#### 4.2.2 PV voltage control loop

The PV voltage loop generates a current reference for the PV current loop. In such control structures, the inner loop (current) presents a delay that cannot be compensated [50]. Therefore the outer loop (voltage) is designed to be slower than the inner loop (current). In this way, one can consider that the inner loop tracks perfectly the reference generated by the outer loop. Assuming this, the plant transfer function is:

(28) $$G_{\mathrm{Vpv}}=\frac{i_{\mathrm{PV1}}}{v_{\mathrm{PV}}}=\frac{-sC_{\mathrm{in}}}{G_{\mathrm{vsensor}}}$$

where $G_{\mathrm{vsensor}}=61$ is the gain of the voltage sensor and $C_{\mathrm{in}}=100$ μF. This only takes into account the input capacitor for the PV port of the converter. The current coming from the PV panel will increase as the maximum power point (MPP) is reached from open-circuit voltage. This means that the expected time to reach the voltage reference will be strongly influenced by the current capability of the connected PV panel. The chosen voltage controller comprising a high-frequency pole and an integrator is:

(29) $$C_{\mathrm{Vpv}}=\frac{2\pi 2720}{s+2\pi 2720}\frac{2\pi 3.5}{s}$$

Obtained response takes about 0.5 s to reach the reference value after applying a step. This is faster than the MPPT algorithm execution period set to 1 s.

#### 4.2.3 Battery current control loop

Battery current control loop is chosen to be 100 times slower than PV current loop. This will allow the PV current loop to adapt the duty cycle $D_{1}$ (and $D_{1}^{\prime }$) while $D_{3}$ and $D_{2}$ (and $D_{2}^{\prime }$) are changing.

The transfer function relating the change of the battery current to the change of $d_{3}$ or $d_{2}$ is just a gain that equals $2I_{L}$ adding a negative sign for $d_{2}$. In order to design a current controller, an I type compensator is used together with a high-frequency pole. The I compensator will introduce a −20 dB/dec slope at crossover frequency $f_{\mathrm{c3}}=f_{c-\mathrm{PV}}/100=27.2$ Hz and the high-frequency pole will further attenuate high frequencies. High-frequency pole is placed at $f_{\mathrm{HFpole3}}=60f_{\mathrm{c3}}$ and the I compensator pole is placed at:

(30) $$f_{\mathrm{Icomp}}=\frac{f_{\mathrm{c3}}}{{\left\|T_{\mathrm{u3}}\right\|}_{f_{\mathrm{c3}}}}$$

where ${\left\|T_{\mathrm{u3}}\right\|}_{f_{\mathrm{c3}}}$ is the gain of the uncompensated loop $T_{\mathrm{u3}}$ at $f_{\mathrm{c3}}$.

**Figure 11:**
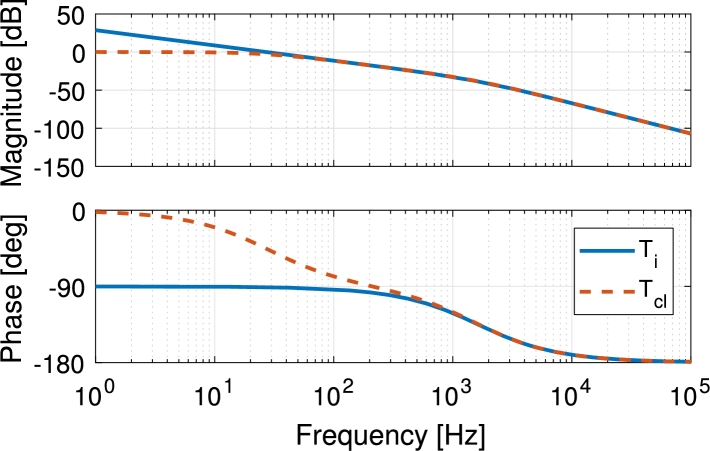
Calculated frequency response of the open- (solid blue) and closed-loop (dashed orange) compensated battery discharge current-loop.

In Figure 11 the calculated open- ($T_{i}$) and closed-loop ($T_{\mathrm{cl}}$) response of the battery discharge current loop is shown at $I_{L}=2$ A. Using this current controller the loop has a high DC-gain, high phase margin $\phi_{m}=89{}^{\circ}$ and achieved a fast response without compromising the PV current tracking and without overshoot even at different operating points (different $I_{L}$). In order to use the same compensator for battery charge, a gain of 2 is added to compensate for the different PWM gain.

## 5 Results

**Figure 12:**
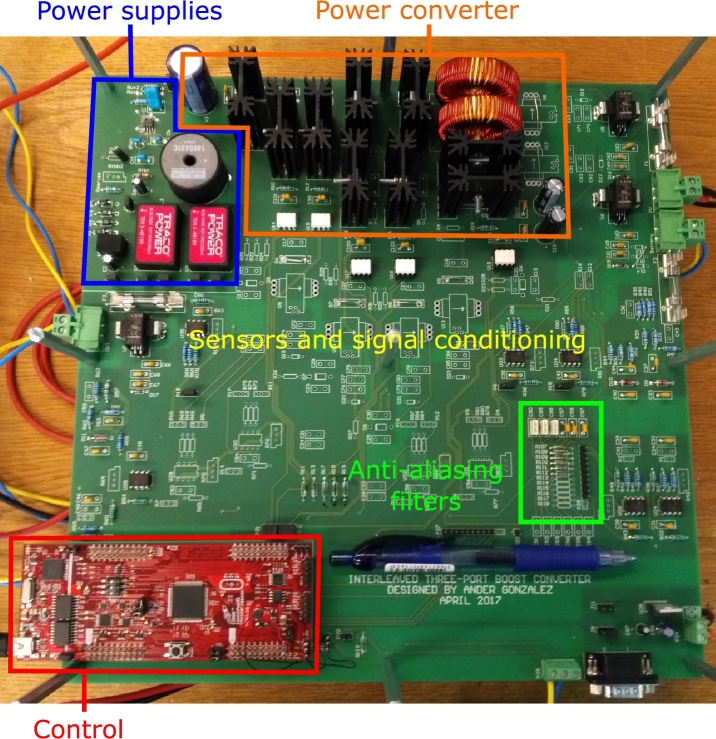
Laboratory prototype of the interleaved three-port boost converter.

Simulation and experimental results are obtained using a PV voltage equal to 32 V and a load resistor of 33Ω connected to the output port. The converter prototype used in order to obtain experimental results is shown in Figure 12.

**Figure 13:**
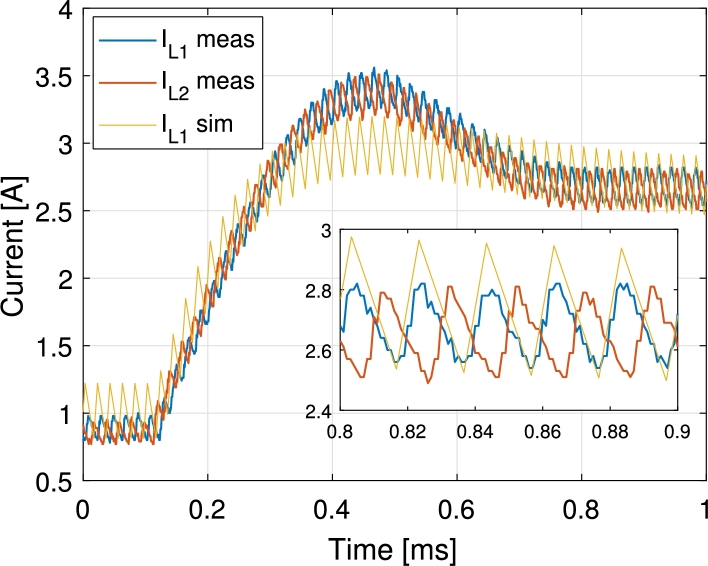
Measured inductor currents and simulated inductor *L*_1_ current during PV current reference step change from 2 A to 5.5 A. The PV current is equally shared by the two inductors.

**Figure 14:**
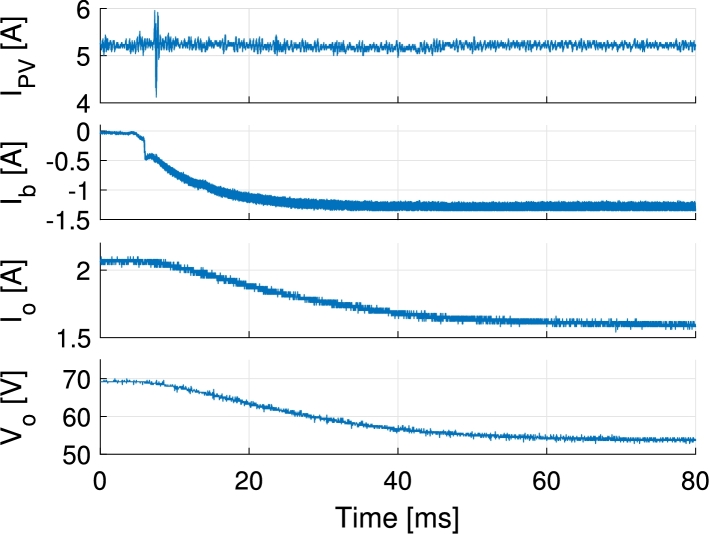
Measurements of PV current, battery current, output current and output voltage during a battery current step change from 0 A to −1.2 A (charge) while PV current is 5.2 A.

**Figure 15:**
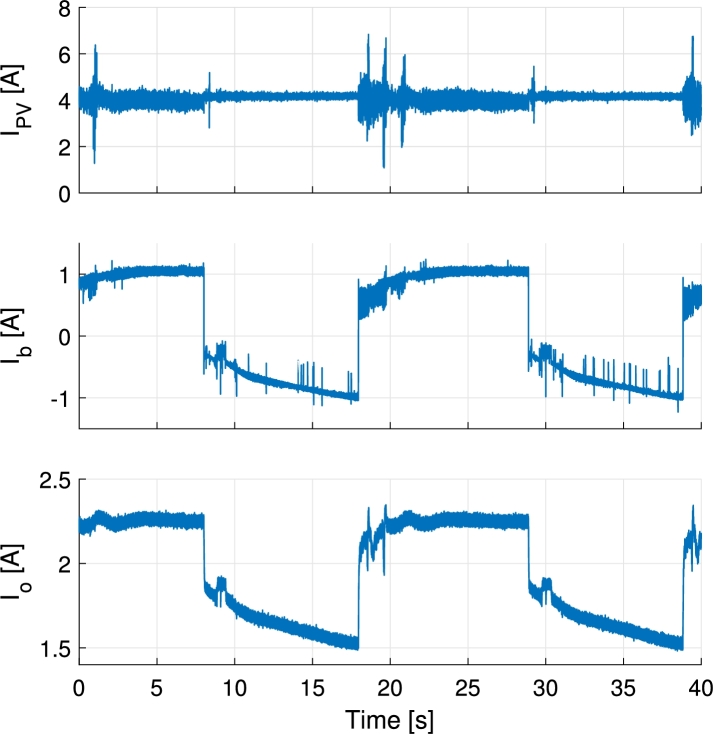
Measurements of PV, battery and output currents for battery charge/discharge transitions applying ±1 A reference, controlling PV current to 4 A and connecting the output to a 33Ω resistive load.

**Figure 16:**
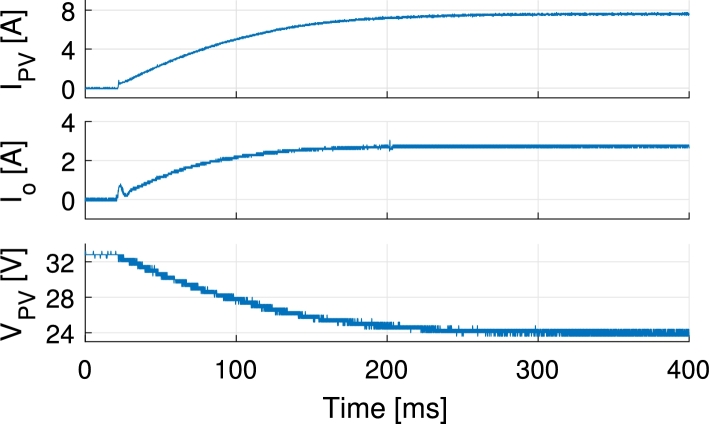
Measurements of PV current, output current and PV voltage during PV voltage reference step change from 34 V (zero current) to 24 V (previously identified MPP) using a Benq GreenTriplex PM245P00 PV panel. Output voltage is held constant to 60 V.

In Figure 13 the inductor currents are shown for a PV current reference step change from 2 A to 5.5 A. The step response exhibits an slightly greater overshoot than the one expected from simulations and control design. This is due to the delays introduced during the digital implementation of the control loops. During the control loop design the PWM delay, control loop execution delay and the anti-aliasing filter that is placed before the ADC have been neglected. These delays add up and deteriorate the response of the system. In Figure 14 all port currents and the output voltage are shown during a battery current step change from 0 A to −1.2 A (charge) while PV current is kept at 5.2 A. There it is appreciated how the PV current loop keeps the PV current at the specified point while the current is partially redirected from the output port to the battery port. In Figure 15 the PV, battery and output currents are shown for transitions from battery charge to discharge and vice versa. During this test the PV current is controlled at 4 A and the output port is connected to a 33Ω resistive load. The PV and battery ports are held to constant 32 V and 48 V respectively. As it can be appreciated, the PV current controller has to adapt the duty cycles of $S_{1}$ and $S_{1}^{\prime }$ when battery is discharged. This is a consequence of changing the duty cycle of $S_{3}$ and generates a short time perturbation in the PV current. Ways to lower the effect of this are making the PV current controller faster, or making the battery discharge controller slower. For the application proposed a slow controller is preferred, as the changes in output power of the modules in a distributed MPPT system may affect other modules in the same string. The test shown in Figure 15 is for illustrative purposes. This type of transitions will not be likely to happen with the proposed control and these are more common when the output voltage is controlled. In order to better perform in controlling the output voltage a redesign of the bandwidth of the controllers, or using different control structures such as the one presented in [51] is suggested. In Figure 16 PV voltage and current and output current are shown for a PV voltage reference step change. The output of the converter is connected to a 60 V power supply. PV voltage reference is higher than the actual voltage in the beginning, thus no current is supplied by the PV panel. Then a step change is applied to the reference down to 24 V, that corresponds to the previously identified MPP voltage of the panel.

**Figure 17:**
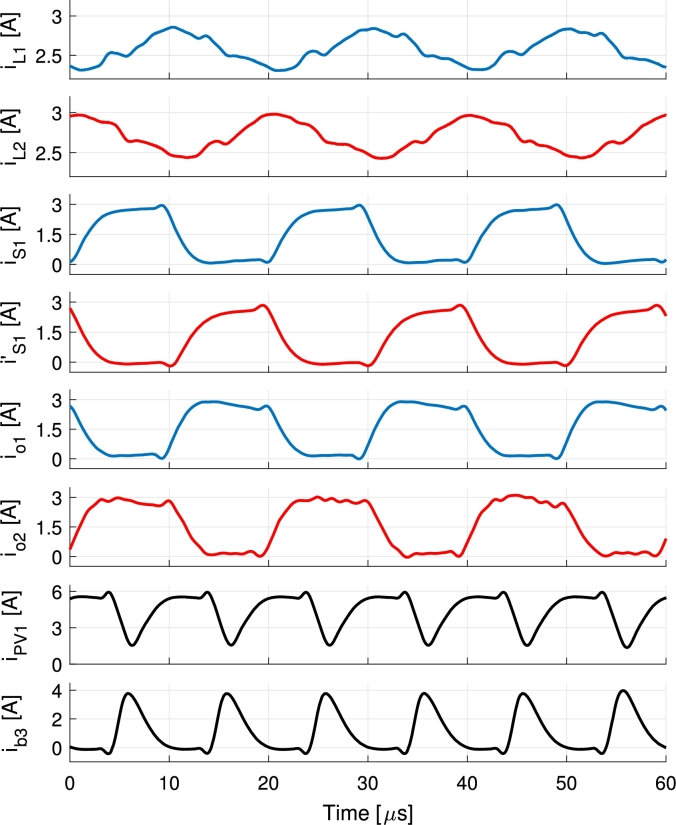
Measurements of the current waveforms of the converter during battery discharge for *I*_PV_ = 4 A, *I*_b_ = 1 A with port voltages held to *V*_PV_ = 32 V, *V*_b_ = 48 V and *V*_o_ = 60 V. Output port supplies a programmable load. The currents of the first branch are represented in blue, second branch in red and the waveforms concerning both branches in black.

**Figure 18:**
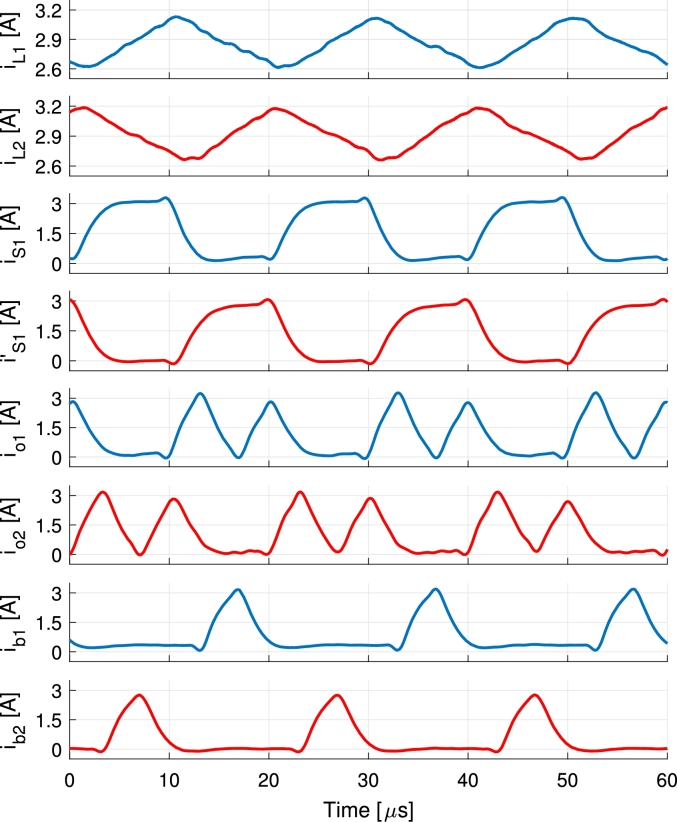
Measurements of the current waveforms of the converter during battery charge for *I*_PV_ = 5.5 A, *I*_b_ = −1 A with port voltages *V*_PV_ = 32 V, *V*_b_ = 48 V and *V*_o_ = 61 V. Output port supplies a 33Ω resistive load. The currents of the first branch are represented in blue and the currents of the second branch in red.

The current waveforms of the converter are shown in Figures 17 and 18 for battery discharge and charge operations respectively. The currents of the first branch are represented in blue, second branch in red and the waveforms concerning both branches in black. Measurements are performed using Tektronix A622 current probes and a PicoScope 4824 oscilloscope. Discharge is performed holding the port voltages to $V_{\mathrm{PV}}=32$ V, $V_{b}=48$ V and $V_{o}=60$ V and controlling the currents to $I_{\mathrm{PV}}=4$ A and $I_{b}=1$ A values. Charge is performed applying $V_{\mathrm{PV}}=32$ V and $V_{b}=48$ V port voltages, connecting a 33Ω load to the output and controlling the PV and battery currents to 5.5 A and −1 A respectively. The resulting output voltage is 61 V. In Figure 18 a further advantage of the modulation is shown. The current pulses feeding the output $i_{\mathrm{o1}}$ and $i_{\mathrm{o2}}$ are split in two rather than being a solid single pulse of current per period. This occurs due to the deviation of the current in order to charge the battery, that happens in the middle of the pulse of current directed to the output port. This can be clearly appreciated when compared to the same currents in Figure 17 and it further decreases the output voltage ripple in the converter. In Figure 17 currents $i_{\mathrm{PV1}}$ and $i_{\mathrm{b3}}$ are shown. These currents become highly pulsating when PV and battery powers are used to supply the output port. During this operation the PV current $i_{\mathrm{PV}}$ and battery current $i_{b}$ stay smooth thanks to the capacitors placed in each port, $C_{\mathrm{in}}$ and $C_{b}$, that filter out all the current pulses ($i_{\mathrm{PV1}}$ and $i_{\mathrm{b3}}$). For the same reason, although the currents $i_{\mathrm{b1}}$ and $i_{\mathrm{b2}}$ shown in Figure 18 are pulsating, the battery current $i_{b}$ does not present a significant ripple.

**Figure 19:**
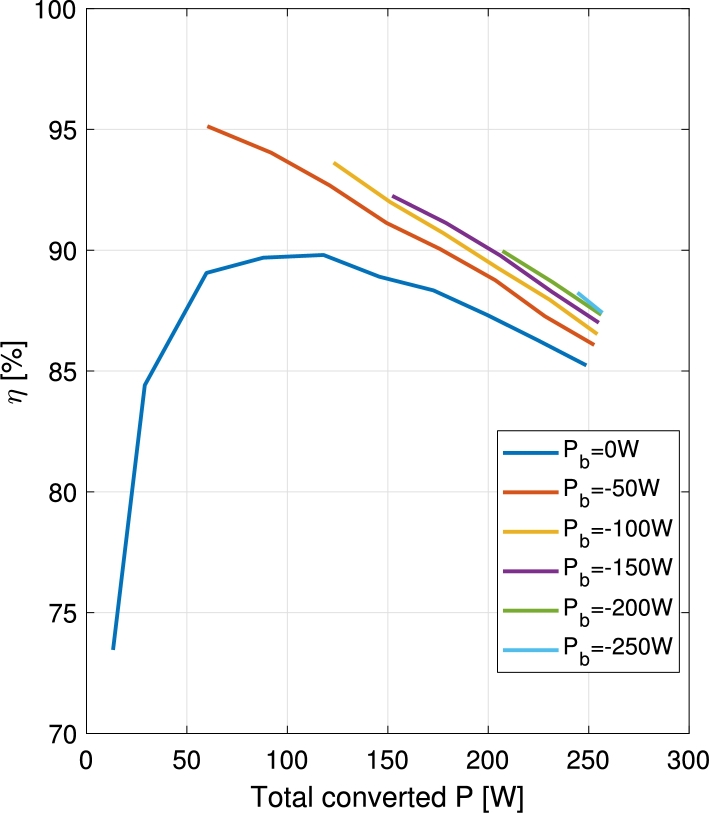
Efficiency vs. total converted power (*P*_o_ − *P*_b_) curves for constant battery charge power.

**Figure 20:**
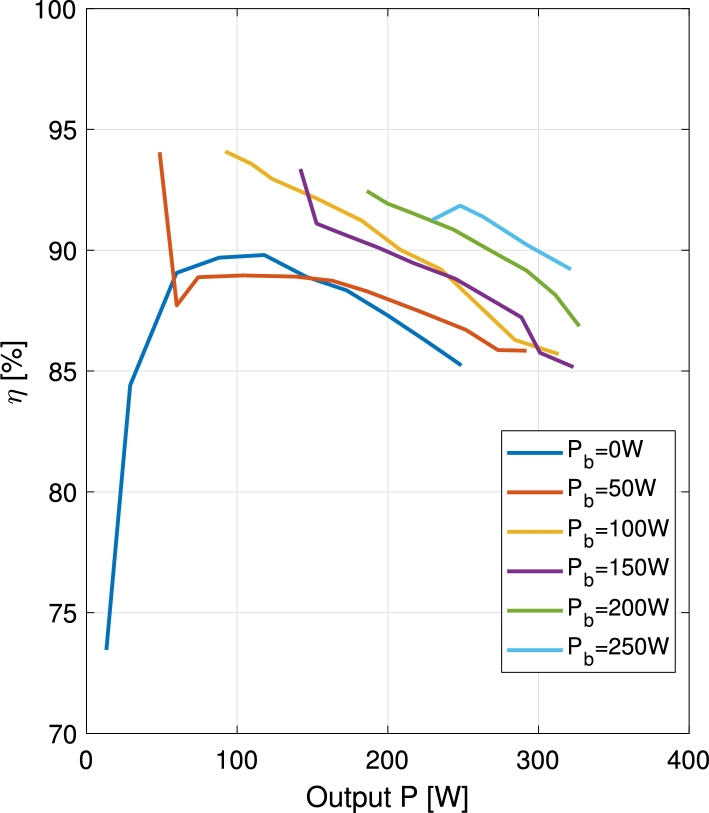
Efficiency vs. output port power (*P*_o_) curves for constant battery discharge power.

The efficiency of the converter has been measured for all the different operation modes. In Figures 19 and 20 the efficiency of the converter is shown for charge and discharge operations respectively. The efficiency is plotted versus the total converted power, namely the sum of the powers supplied to all the ports that behave as an output. During the battery charge operation, the power supplied to the battery is subtracted to the power supplied to the output port, while during the battery discharge, all the converted power is supplied to the output port. Measurements are taken for $V_{\mathrm{PV}}=32$ V, $V_{b}=48$ V, $V_{o}=60$ V, and the following limits: $P_{\mathrm{PV}}<275$ W and $P_{\mathrm{Tot}}<350$ W.

In both cases, when charging or discharging the battery, the efficiency improves with respect to the case when the battery is not used. This can be explained with the voltage differences in the ports. When the battery is charged, part of the PV power is diverted to the battery port, instead of to the output port. In this case, as the battery port shows a lower voltage than the output port, the differences in input and output voltage are reduced. When the battery is discharged, part of the supply is given by the battery, instead of the PV port. In this case, as the battery shows a higher voltage than the PV port, the difference in voltages between input and output ports of the converter is again reduced. Since the converter is in essence a modified boost converter, it benefits from lower voltage differences between its input and output ports, thus increasing the efficiency during these two operations when compared to batteryless operation.

In Figure 20, in the battery discharge curves, a sudden drop in efficiency occurs between the first and the following recorded points. This can be clearly noticed in the curves corresponding to 50 W and 150 W. The drop is provoked by a passive snubber circuit included in the $D_{\mathrm{PV}}/S_{3}$ switching cell. In the first point of the curve $S_{3}$ is permanently closed, being the battery the sole power supplier. In the following points, the $D_{\mathrm{PV}}/S_{3}$ cell is switching, further contributing to the losses.

**Figure 21:**
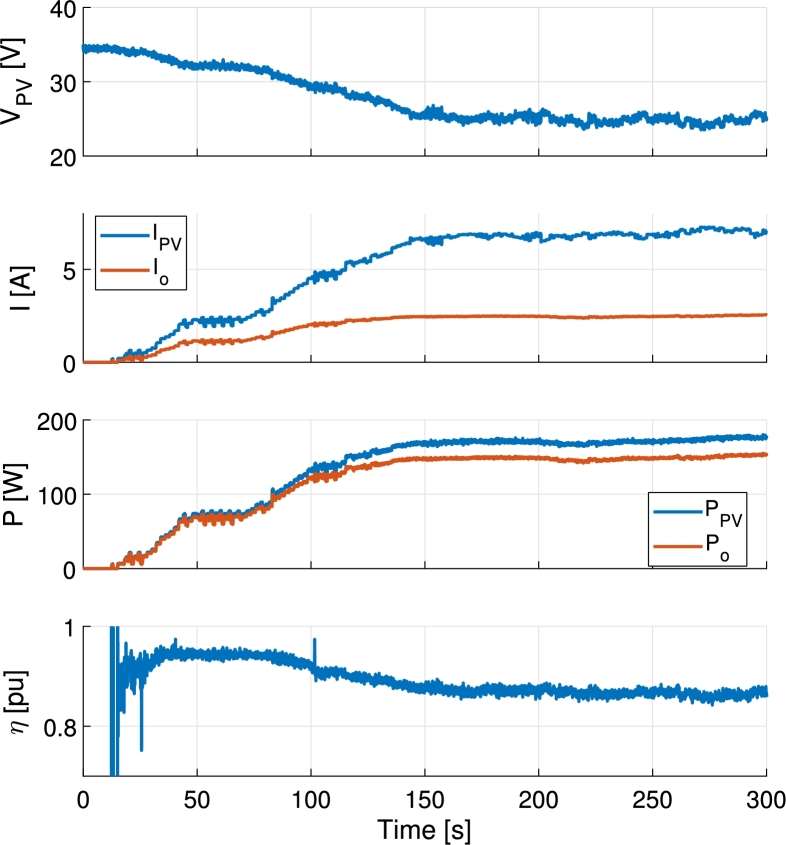
MPPT of a Benq GreenTriplex PM245P00 PV panel from stopped operation. Output voltage *V*_o_ = 60 V.

In Figure 21 the MPPT operation, starting from stopped operation, is shown. The selected MPPT algorithm is of perturb and observe (P&O) type. The algorithm is executed once every second and applies a new PV voltage reference. The latter is calculated by applying a voltage step of 0.2 V to the actual value of the PV voltage read by the converter. During this test, the output voltage $V_{o}$ is kept constant at 60 V and the battery port is not used. The selected PV panel is a Benq GreenTriplex PM245P00. The data is logged using LabVIEW and a national instruments niDAQ system working at 50 ms sampling period. The efficiency *η* is calculated dividing the output port power, $P_{o}$, by the PV power, $P_{\mathrm{PV}}$. This may cause some punctual error during fast current changes in the calculation of the efficiency.

## 6 Conclusion

In this paper an in-depth analysis of the interleaved three-port boost converter is presented.

The presented symmetrical modulation allows for sampling of the average value of the current. Besides, the sampling never occurs while switching is undergoing. This improves data measurement and thus reference tracking. The modulation is validated using the experimental setup, where the correct scheduling of the switch ON-states is verified.

During control loop design, modeling of the effects that appear due to digital implementation (i.e. PWM modulation, control loop execution delay, etc.) should not be neglected if an accurate waveform shape of the response is required. Usually this can be worked around by designing the control loop to have a high enough phase margin.

It is demonstrated that choosing the proper bandwidth of the controller the different current loops can be treated separately achieving good performance.

The controller design procedure is demonstrated to be valid if the right working points are selected. System offers good response even in the vicinity of the selected points, being able to control the PV panel voltage and current and battery current.

The interleaved three-port boost converter achieves very low current and voltage ripples for any of the conversion paths, even at high power. This makes the converter suitable for a grid-tied distributed MPPT system including storage at module level.

The MPPT tracking of the system is done by PV voltage reference setting. The applied step will determine the amount of power increment during the steps. A high power increment may disturb the proper working of a series connected distributed MPPT system. Here a small voltage step, of 0.2 V, is proposed in order to keep the power increment moderate. This step is enough to reach the MPP in a reasonable time while keeping the distributed MPPT system within safe operation area.

## Declarations

### Author contribution statement

Ander González: Conceived and designed the experiments; Performed the experiments; Analyzed and interpreted the data; Contributed reagents, materials, analysis tools or data; Wrote the paper.

Ramón López-Erauskin: Analyzed and interpreted the data.

Johan Gyselinck: Contributed reagents, materials, analysis tool or data.

### Funding statement

This work was supported by the Belgian Walloon region under project BATWAL, convention number 1318146.

### Competing interest statement

The authors declare no conflict of interest.

### Additional information

No additional information is available for this paper.
